# Improving the Value Utilization of Tuna Peptide Powder for the Cosmetics Field Through Ozone Oxidation

**DOI:** 10.3390/md23050191

**Published:** 2025-04-28

**Authors:** Haolong Zheng, Shiyang Gu, Shiqi Huang, Yan Zhang, Feng Xu, Daofei Lv, Wenbing Yuan, Kongyu Zhu, Xin Chen

**Affiliations:** School of Environmental and Chemical Engineering, Jiangwan Campus, Foshan University, Foshan 528000, China; zhl000507@163.com (H.Z.); 17841341514@163.com (S.G.); huangski@163.com (S.H.); fengxu@fosu.edu.cn (F.X.); lvdaofei@fosu.edu.cn (D.L.); hnyuanwb@126.com (W.Y.); zhukongyu163@163.com (K.Z.)

**Keywords:** tuna peptide, deodorization, ozonation, cosmetics

## Abstract

The existing in vitro and clinical trial evidence supports the health and wellness benefits of collagen peptides sourced from various origins. Despite this, research on collagen peptides from tuna remains limited. Notably, tuna-derived peptides possess an inherent fishy odor, rendering them unsuitable for direct application in humans. This study explores the enhancement of tuna peptides’ applicability in cosmetics through odor mitigation. We developed a dual-phase ozone treatment, employing both dry and wet ozone, to deodorize tuna peptide powder, enabling its use in cosmetic formulations. The deodorized tuna peptide powder can be used in cosmetics. We optimized the ozone nitrification and deodorization conditions for tuna peptide powder by adjusting the treatment time, ozone concentration, and temperature. Sensory evaluation and GC-MS analysis confirmed the effectiveness of fishy odor removal, offering a comprehensive understanding of the deodorization process. The findings reveal that wet ozonation at 50 °C with an ozone concentration of 99.1 mg/L for 40 min significantly reduces the fishy odor of tuna peptide powder. Notably, n-Hexaldehyde, the primary odor-contributing volatile compound, decreased by 66.5%, confirming the efficacy of ozone treatment in odor mitigation. Moreover, the protein activity within the powder remained unaffected, ensuring the preservation of its functional properties. This study demonstrates the efficacy of ozone oxidation in adapting tuna peptide powder for cosmetic use.

## 1. Introduction

Marine biological peptides are naturally active peptides extracted from marine organisms, possessing a diverse range of physiological functions and benefits. *Bonito* and *yellowfin tuna* peptide powders offer a range of advantages, from nutritional benefits to potential applications in cosmetics [1]. Tuna collagen peptides are receiving increasing attention as a source of protein due to their availability, environmental safety, and ability to circumvent religious restrictions and health concerns associated with swine flu and bovine spongiform encephalopathy [2,3]. The in vitro bioactivity effects of tuna collagen peptides were evaluated in terms of collagen and elastin synthesis and senescent cell inhibition against human dermal fibroblasts [4]. According to Amnuaikit et al. [5], cosmetic products containing marine biological peptides have the ability to enhance facial skin moisture, minimize pores and skin wrinkles, and significantly brighten the skin when used regularly for a minimum of two weeks. It is 100% absorbable through the skin [6].

However, the fishy odor of tuna peptide powder does not allow it to be used directly in cosmetics. Fishy odor is one of the major factors affecting the quality of fish and their acceptance by consumers [7,8,9]. The main methods of deodorization currently used are described below.

Masking, one of the earliest deodorization techniques, employs volatile gases from other substances to conceal the inherent fishy odor [2]. However, this method fails to eliminate the original odor entirely and introduces additional compounds. Zeng et al. used selective adsorbents such as activated carbon and zeolite to adsorb tilapia enzyme digests, which ultimately effectively removed the fishy flavor and retained relatively high protein levels [10]. However, this absorbent was responsive to different removal gases. This approach effectively removed the fishy odor while preserving a high protein content. Nonetheless, the adsorbent’s efficacy varied with different gases. Adsorbent options are restricted, and high-cost, saturated adsorbents require subsequent treatment to mitigate environmental pollution and adverse effects. Wu et al. demonstrated that adding 1.5% cyclodextrin β to eel meat and stirring for 90 min significantly diminished the odor [11]. Although effective to some extent, this method fails to entirely eliminate volatile odors, with notably poor encapsulation of macromolecular volatiles. Fu et al. employed an alkaline treatment on silver carp [12]. This significantly reduced the ichthyoid content in the water but also degraded the nutrients of the aquatic product. The residual alkalinity in the sample was difficult to remove, necessitating treatment of the waste liquid prior to discharge. In biological deodorization, specific microorganisms are introduced under controlled temperature and humidity to utilize microbial metabolites for reducing odorous substance concentrations. Xu et al. conducted the fermentation of silver carp surimi using yeast at 35 °C for 1.5 h [13], which effectively diminished the fishy odor. However, this approach is limited in its applicability, as different fish species show varying resistance to the microorganisms, and prolonged treatment periods can lead to the generation of by-products through microbial activity.

Ozone oxidation is advantageous for deodorizing polymer-active peptide ingredients in cosmetics [14]. Ozone, characterized by a redox potential of 2.07 V, is a potent oxidizing agent, surpassed only by fluorine in terms of oxidative strength. Ozone interacts with aldehydes, alcohols, amines, and other volatile compounds in aquatic products to diminish or remove odorous substances, thereby achieving deodorization [15]. Following treatment with floating ozone technology, the volatile component levels in silver carp minced meat are significantly reduced without the introduction of additional odors [16]. In contrast, the use of ozone oxidation technology for removing the fishy odor of tuna peptide powder offers several advantages: (1) Moisturizing capacity—marine peptides extracted from tuna organisms have excellent moisturizing ability. After ozone treatment, these peptides may exhibit better hydrophilicity, thus enhancing their ability to absorb and retain water in the skin [17]. These excellent moisturizing abilities are essential for maintaining skin elasticity and preventing skin dryness [18,19]. (2) Antioxidant properties—marine peptides, particularly those derived from fish and shellfish, have been demonstrated to stimulate fibroblast activity, which is crucial for collagen synthesis. Collagen represents the primary structural protein in the skin, and its production declines with age [20]. By stimulating collagen synthesis, ozone-treated marine peptides can assist in maintaining skin elasticity and reducing the appearance of wrinkles [21,22]. (3) Wound healing and anti-inflammatory properties—the available evidence indicates that marine peptides have the potential to accelerate the healing of wounds and exert anti-inflammatory effects. It is postulated that the application of ozone treatment may serve to enhance these properties, thereby rendering these peptides suitable for incorporation into formulations designed to provide soothing relief to irritated skin and accelerate the healing process [23,24].

Therefore, the purpose of this study is to utilize ozone oxidation to create a deodorizing process suitable for tuna polymer peptide powder in the field of cosmetics. After deodorization, the tuna peptide was assessed by sensory evaluation, gas chromatography–mass spectrometry (GC-MS), cold/heat stability analysis, the determination of amino nitrogen and total nitrogen content before and after oxidation, and other methods to confirm the comprehensive feasibility of the process conditions.

## 2. Results

### 2.1. The Optimum Conditions for the Dry Ozonation

The duration of dry ozonation had a small impact on the deodorization of the samples, while the ozone concentration significantly affected the sensory scores. The average sensory score was 4.5 at ozone concentrations of 0.1 mg/L–50.2 mg/L, which decreased to 3.7 when the ozone concentration was increased to 99.1 mg/L and significantly decreased to 3.5 at a concentration of 104.6 mg/L (*p* < 0.05). In orthogonal dry ozonation experiments, under 60 min treatment, the sample with an ozone concentration of 99.1 mg/L had a sensory score of 4.2 ± 0.4, which was significantly higher than 3.4 ± 0.2 (*p* < 0.05). The optimal dry ozonation conditions for tuna peptide powder were determined to be an ozone concentration of 99.1 mg/L with a treatment duration of 180 min (Table 1).

### 2.2. The Optimum Conditions for the Wet Ozonation

Tuna peptide powder was treated by wet ozonation in 17 single-factor experiments, and the sensory evaluation results (fishy odor/taste) are shown in Table 2. When the ozone concentration was gradually increased from 0.1 mg/L to 104.6 mg/L with the same reaction time and temperature, the sensory scores ranged significantly from 2.2 to 5.0 (*p* < 0.05). The results obtained in the single-factor experiments were used to design 25 orthogonal experiments of wet ozonation. In orthogonal wet ozonation experiments, the sample with an ozone concentration of 50.2 mg/L had a sensory score of 4.7 ± 0.2, which was significantly higher than 2.3 ± 0.2 (*p* < 0.05). The optimal conditions for the wet ozonation of tuna peptide powder were identified as an ozone concentration of 99.1 mg/L, a treatment duration of 40 min, and a temperature of 50 °C (Table 2).

**Figure 1:**
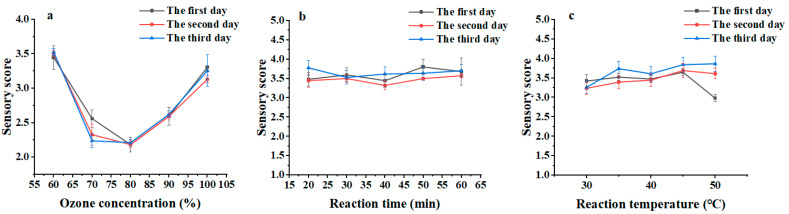
Effect of ozone concentration on sensory score (**a**). Effect of reaction time on sensory score (**b**). Effect of reaction temperature on sensory score (**c**).

The sensory score first decreased and then increased with increasing ozone concentration (Figure 1a). When the ozone gas concentration was increased (the intake flow rate remained unchanged), the concentration of ozone dissolved in the peptide powder solution after a short period increased, thereby increasing the mass transfer efficiency. The sensory score was the lowest (2.2) at an ozone concentration of 99.1 mg/L and then increased again upon further increasing the ozone concentration. The peptide powder solution had reached saturation at an ozone concentration of 99.1 mg/L. When the ozone concentration increased beyond 99.1 mg/L, excess ozone would introduce other odors, reducing the deodorizing effect.

The sensory score changed only slightly with increasing reaction time, producing a curve that approximates a straight line (Figure 1b). The sensory score changed only slightly with increasing reaction temperature, also producing a curve that approximates a straight line (Figure 1c). Although the increase in the temperature of the peptide powder solution increased the activity of organic molecules, it also decreased the solubility of ozone and thereby reduced the mass transfer efficiency. As a result, the impact of increasing temperature on the deodorizing effect was reduced. Therefore, the reaction temperature also had only a small effect on the deodorizing effect of wet ozonation.

The data presented in Table 2 and Figure 1 show that, of the three variables, ozone concentration has the greatest impact on the deodorizing effect of wet ozonation of tuna peptide powder, with reaction temperature and reaction time having only a small impact. The optimal conditions for treating tuna peptides by wet ozonation were as follows: ozone concentration, 99.1 mg/L; reaction time, 40 min; and reaction temperature, 50 °C.

### 2.3. Low Loss of Total Nitrogen and Amino Acid Nitrogen

The effects of dry and wet ozonation on total nitrogen and amino nitrogen content in tuna peptide powder were explored, as shown in Table 3. The wet sample’s total nitrogen content (15.0 ± 0.2) showed no significant difference from the original sample. In terms of amino acid nitrogen (g/100 g), the original sample (2.5 ± 0.1), dry sample (2.2 ± 0.2), and wet sample (2.3 ± 0.1) exhibited no significant differences (*p* > 0.05). This demonstrates the stability of the deodorizing effect of ozonation on tuna peptide powder and supports their use as an ingredient in cosmetics.

### 2.4. High Stability at Low and High Temperatures

The qualitative sensory scores of cosmetic samples containing 1% ozone-treated tuna peptides remained unchanged in the low- and high-temperature stability tests (Table 4). A moderately acceptable sensory score is 2–3. A barely acceptable sensory score is 3–4. An unacceptable sensory score is 4–5. The cosmetic containing 1% untreated tuna peptide powder exhibited rapid sensory deterioration upon returning to room temperature, with scores transitioning from “barely acceptable” on day 1 to “unacceptable” from day 3 to 28. In contrast, cosmetics containing 1% ozone-treated tuna peptide powder consistently maintained a “moderately acceptable” rating throughout the 28-day test period, with no observable increase in fishy odor or off-flavors. In other words, the fishy odor/taste of the samples did not increase due to changes in temperature. Moreover, the ozone-treated samples were more sensorily acceptable than the untreated samples. This demonstrates the stability of the deodorizing effect of ozonation on tuna peptide powder.

### 2.5. Reduction in Fishy Odor

GC-MS analysis revealed that the original concentration of n-hexaldehyde was 5.9 ± 0.2 μg/L, which decreased significantly to 2.0 ± 0.1 μg/L following ozone treatment (*p* < 0.05), representing a reduction of 66.5 ± 0.2% (Table 5). N-hexaldehyde initially exhibited a markedly high OAV = 295, indicating its dominant sensory contribution. Following ozonation, this value was substantially reduced to 100. In contrast, the OAV for nonanal transitioned from below the typical sensory relevance threshold (0.7) to slightly above (1.6), while the OAV for octanal remained negligible throughout the process (increasing from 0.06 to 0.1). Crucially, although the OAV of nonanal marginally exceeded the sensory threshold after ozonation, its overall contribution to the aroma remained minor compared to n-hexaldehyde, whose OAV remained around 60-fold higher. These huge differences collectively underscore the role of n-hexaldehyde as a primary contributor to the characteristic fishy odor.

In addition, compounds recognized in the literature as significant contributors to fishy odor [25] were detected in our samples. A comparative analysis of GC-MS peak areas for a range of such potential odorants (including the various aldehydes, alcohols, sulfur compounds, esters, ketones, and alkanes detected) before and after treatment is provided for reference in the Supplementary File (Table S1). Among the C8 compounds, 2-ethyl-1-hexanol and butyl butyrate were associated with mild floral/oily and fruity notes, respectively. Additionally, C7 compounds benzaldehyde (almond aroma) and 3-heptanone (fruity/ketonic) were present. The detection of dimethyl trisulfide is noteworthy, as sulfur compounds often possess low odor thresholds and can significantly impact odor profiles [26].

## 3. Discussion

O_3_ shows different deodorizing effects, with several factors influencing its removal efficacy [27]. The findings of the analysis demonstrate that the ozone concentration exerts a more significant influence on the deodorization effect than the reaction time and ozone concentration in both the dry ozone and wet ozone methods. These results are consistent with the findings of Wang et al., whereby ozonated water had the best deodorizing effect on minced silver carp at an initial concentration of 0.78 mg/L [5]. Therefore, increasing the ozone concentration effectively improved the deodorizing effect of dry ozonation. Under optimal conditions, a minimum sensory score of 2.3 was achieved, reflecting a 54% reduction, comparable to the significant decrease of 50.2 mg/L reported by Chen et al. [28] in deodorizing sea bass meat with a combined method and the 46% reduction by Wang et al. in deodorizing minced silver carp with ozonated water. The alterations in total nitrogen and amino nitrogen content were minor relative to the previously documented 22.9% nitrogen loss in Antarctic krill meal treatment [29]. Manouchehr et al. [30] observed that volatile aldehydes are recognized for generally possessing very low odor thresholds, enabling them to significantly influence aroma profiles even at minimal concentrations. Our results show that n-hexaldehyde exhibited markedly higher Odor Activity Values (OAVs) than nonanal and octanal, confirming its dominant contribution to the perceived odor profile. Consequently, the substantial 66.5% depletion of n-hexanal achieved via the optimized ozone treatment directly correlates with, and is considered primarily responsible for, the significant decline in fishy odor observed during sensory evaluations (Table 4). This observation is consistent with the understanding that short-chain aldehydes, such as n-hexaldehyde, typically impart pungent or characteristic off-notes significant to perceived quality [30]. Furthermore, the contact area and pH also impact the effectiveness of ozone deodorization; however, the literature suggests these factors have minimal influence on the deodorization outcome [31].

Ozone oxidation manifests in two forms: direct and indirect oxidation. Ozone oxidation manifests in two forms: direct and indirect oxidation. Direct oxidation functions via a mechanism that typically transpires in solutions laden with free radical terminators, such as carbonic acid. This oxidation can convert organic material into simpler entities like carboxylic acids or fully oxidize it to carbon dioxide and water [32]. Ozone directly oxidizes peptides by targeting specific amino acids, particularly sulfur-containing residues (cysteine and methionine) and aromatic residues (tryptophan, tyrosine, and histidine), leading to oxidation products such as trisulfides (dimethyl trisulfide) and oxidized aromatic derivatives (benzaldehyde and dibutyl phthalate) [33]. Notably, during direct oxidation, ozone rapidly reacts with unsaturated compounds, including double bonds and aromatic compounds endowed with electron-donating substituents such as phenolic hydroxyl groups [34].

Wet ozone oxidation employs an indirect mechanism where, in the presence of free radical activators and promoters, ozone generates a significant number of free hydroxyl radicals within the reaction system. These radicals initiate a chain reaction that produces additional reactive radicals. The hydroxyl radical (-OH) produced in the indirect oxidation process serves as the best oxidant for deep oxidation, which rapidly oxidizes the organic matter and reduces the organic carbon content in water [35]. Indirect oxidation may lead to notable secondary structural modifications in proteins [36,37]. Although this study did not investigate peptide structure in detail, it is important to note that our compositional analysis showed stable levels of total nitrogen and amino nitrogen following wet ozone treatment (Table 3), indicating that the core peptide structures and their primary amino groups remained largely unaffected. This suggests the parameters used (high ozone dose, moderate temperature, and short exposure) favored selective oxidation rather than extensive peptide degradation. This outcome aligns with previous studies on the ozone treatment of fish protein [37] and marine peptides, which reported improvements in sensory or functional properties while preserving nutritional and bioactive integrity [37]. Thus, significant destructive oxidation of the target peptides was largely avoided. Further research is needed to confirm these effects in more detail.

The safety of ozone treatment for cosmetic peptides is supported by two factors. First, various ozonized plant oils produced through controlled ozone treatment are already approved as cosmetic ingredients. They are widely used as skin conditioners and have a strong record of safe topical application [38]. Second, ozone breaks down into harmless substances like oxygen and water [39]. Advantageously, this decomposition mechanism intrinsically avoids generating persistent chemical pollutants or harmful residues, thereby significantly minimizing the risks of secondary pollution to human health and the environment.

## 4. Materials and Methods

### 4.1. Experimental Materials

The materials used in this study include Pulvis tuna oligopeptide powder (Liaoning Taiai Peptide Bioengineering Technology Co., Ltd., Dalian, China) and sodium chloride (AR; Aladdin Reagent (Shanghai) Co., Ltd., Shanghai, China). The instruments used include a Kjeldahl nitrogen analyzer (K9840, Hanon Advanced Technology Group Co., Ltd., Jining, China), a freeze dryer (LGJ-10, Sihuan Keyi Technology Development Hebei Co., Ltd., Tangshan, China), an ozone generator (QD-Y, Guangzhou Qida Environmental Protection Equipment Co., Ltd., Guangzhou, China), a heat-collecting thermostatic magnetic stirrer (DF-101S, Gongyi Yuhua Instrument Co., Ltd., Gongyi, China), and ozone concentration detectors (Model 106-H and 202-L, Passkey Technology Co., Ltd., Boulder, CO, USA). To ensure accuracy in our experiment, ozone concentration detectors were used to obtain the ozone concentration [40].

### 4.2. Dry Ozonation of Tuna Peptides

**Figure 2:**
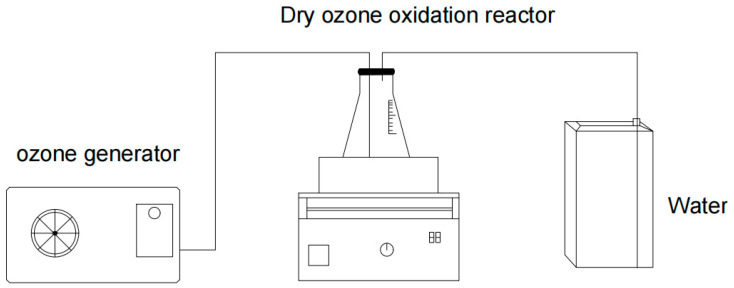
Apparatus for the dry ozonation of tuna peptides.

First, 1 g of tuna peptide powder (±0.05 g) was weighed using an electronic balance. The measured powder was then placed in a conical flask equipped with two tubes and fitted with a rubber stopper. One tube was connected to the ozone generator and the other to a water tank designated for exhaust gas treatment (Figure 2). After connecting the ozone generator water supply device, the main power was turned on and the device was allowed to run for 5 min. Subsequently, the ozone switch was turned on and set to deliver specific ozone concentrations (0.1 mg/L–104.6 mg/L) over a specified period (0–300 min). A consistent ozone flow rate of 1.5 L/min was maintained throughout all experiments. After the ozone supply was switched off, the rubber stopper was removed, and the mixture was allowed to stand for 10 min. When the ozone in the flask had decomposed, the peptide powder was removed for sensory evaluation.

### 4.3. Wet Ozonation of Tuna Peptides

**Figure 3:**
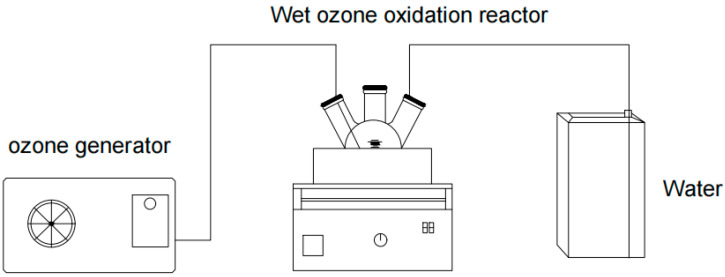
Apparatus for the wet ozonation of tuna peptides.

First, an electronic balance was used to weigh 1 g of tuna peptide powder. Next, a graduated cylinder was used to measure 5 mL of distilled water, which was placed in a three-necked flask. Then, the weighed tuna peptide powder was placed in the three-necked flask containing distilled water, and it was ensured that the rubber stoppers were securely in place on each neck. Then, a tube was inserted through the stoppers on both sides, connecting one end to the ozone generator and the other to the tap water tank for exhaust gas treatment (Figure 3). After connecting the ozone generator water supply device, the main power of the ozone generator was switched on, and it was allowed to run for 5 min before the ozone switch was activated and set to provide specific ozone concentrations (0.1 mg/L–104.6 mg/L) over a specified time (0–300 min). A consistent ozone flow rate of 1.5 L/min was maintained throughout all experiments. After the ozone supply was switched off, the rubber stopper was removed, and the mixture was allowed to stand for 10 min. When the ozone in the flask had decomposed, the peptide powder solution was removed and freeze-dried to obtain a dry peptide powder for sensory evaluation.

### 4.4. Sensory Evaluation

With reference to Khazandi et al. [41], the filtrate was sensorily evaluated using a 5-point scale (1, no fishy odor; 2, slight fishy odor; 3, moderate fishy odor; 4, heavy fishy odor; and 5, very heavy fishy odor). Deodorized tuna was sensorily evaluated using a “fishy score”, which was calculated by summing the weighted scores for fishy odor and fishy taste. The higher the scores, the stronger the fishy odor and fishy taste [42,43]. Fishy odor and taste were scored using a five-point scale: 1, no fishy odor/taste; 2, slight fishy odor/taste; 3, moderate fishy odor/taste; 4, strong fishy odor/taste; and 5, very strong fishy odor/taste. The overall average fishy score V for each sample was calculated as follows:

V = (0.6∑Gi + 0.4∑Ti)/10(1)

where Gi is the fishy odor score assigned to the sample by sensory evaluators in order, Ti is the fishy taste score assigned to the sample by the sensory evaluators in order, and 10 is the number of evaluators. Sensory evaluation was repeated on days 1, 3, and 5 following the above method to obtain three sensory scores (Figure 3). Then, we used SPSS (Version 27; IBM Corp., Armonk, NY, USA) to analyze the sensory score and label the differences in the tables. First, one-way analysis of variance (ANOVA) was used to test the significance of differences between groups. The significance of differences between groups was assessed by analyzing the F-statistic and its corresponding *p*-value. In this study, pairwise comparisons with *p*-values below 0.05 were considered significant. Comparisons with *p*-values greater than 0.05 were deemed not significant [30].

The total nitrogen and amino nitrogen contained in the original tuna peptide powder, dry ozone-treated peptide powders, and wet ozone-treated tuna peptide powders were respectively determined according to the GB5009.5-2016 [44] and GB5009.235-2016 [45] national standards of the People’s Republic of China.

### 4.5. Low- and High-Temperature Stability Tests

Temperature stability tests were carried out on hand cream samples containing 1% ozone-treated tuna peptides. Each sample was separated into two vials (A and B), and each vial was sealed with a clean stopper. Vial A was subjected to a 3-day heating–cooling cycle that was repeated 10 times: the sample was placed in an incubator for 24 h at 40 °C, left at room temperature for another 24 h, and finally placed in a refrigerator at –10 °C for another 24 h. Vial A was observed for oil–water stratification and color changes during this process by visual comparison with vial B [46].

### 4.6. Test Conditions for GC-MS Analysis

The tuna peptide powder with the best sensory evaluation after ozonation was selected for volatile compound analysis using GC-MS. Volatile compounds from the untreated and ozonated tuna peptide samples were analyzed by GC-MS and identified through library matching and retention index confirmation. Specifically, we compared each mass spectrum to entries in established spectral databases (the NIST/EPA/NIH Mass Spectral Library and the Wiley Registry) to assign tentative compound identities [47]. Headspace solid-phase microextraction (HS-SPME) was employed for sample pre-treatment. Briefly, 0.2 g of the sample, 1.44 g of NaCl, and 8 mL of deionized water were mixed, transferred to a headspace vial, and sealed. The vial was incubated at 60 °C with agitation for 15 min to reach equilibrium, followed by adsorption using an activated DVB/CAR/PDMS fiber for 40 min. The extracted volatiles were then desorbed at the GC injection port at 250 °C for 5 min.

GC conditions: An HP-5MS capillary column (30 m × 0.25 mm × 0.25 μm) was used, and the temperature of the KC interface was maintained at 240 °C. He (99.99%) was used as the carrier gas at a flow rate of 1.0 mL/min without diverting. The initial temperature of the column was set at 40 °C, and the holding time was 2 min. The temperature of the machine was raised to 160 °C at 4 °C/min, increased to 250 °C at 8 °C/min, and maintained for 10 min.

MS conditions: Solvent removal time, 1 min; ion source, electron bombardment source (EI); ion source temperature, 230 °C; ionization voltage, 70 eV; scanning mass range, 30–500 *m/z*; interface temperature, 280 °C; four-stage bar temperature, 150 °C. Analysis conditions: carrier gas: high-purity helium (purity ≥ 99.999%), in constant flow mode, with no sample splitting, and a column flow rate of 1.0 mL/min; injection port temperature at 250 °C; ion source temperature at 230 °C; quadrupole temperature at 150 °C; programmed temperature conditions: maintained at 40 °C for 2 min, then ramped at 30 °C/min to 180 °C, and finally ramped at 10 °C/min to 270 °C for 3 min. SCAN mode is employed, covering a scanning range of 45–450 u. Solid phase microextraction conditions: extraction head 30/50 μm DVB/CAR/PDMS, extraction duration of 40 min. Quantitative ion: 57 [47,48].

For additional validation, an alternative extraction method was conducted by dissolving 2.0 g of pre-baked NaCl in 10.0 mL of the sample within a 20 mL headspace vial. The mixture was incubated at 50 °C with agitation at 450 rpm for 40 min before GC-MS analysis. The column temperature program included an initial hold at 40 °C for 2 min, followed by a ramp at 30 °C/min to 180 °C, and a final increase at 10 °C/min to 270 °C for 3 min. Retention times and parameters for key olfactory compounds and internal standards are summarized in Table 6 [49].

To evaluate the sensory impact of individual volatile compounds identified by GC-MS, the Odor Activity Value (OAV) was calculated [50]. The OAV for each target aldehyde was determined using the formula: OAV = C/OT, where ‘C’ is the concentration of the compound measured by GC-MS and ‘OT’ is the odor threshold of that compound, typically in water. Odor threshold values for n-hexaldehyde, nonaldehyde, and octanal were obtained from published literature and are listed in Table 5 [51]. A compound is generally considered to make a significant contribution to the overall odor profile when its OAV is greater than 1.

## 5. Conclusions

This study investigated the deodorization effect of ozone oxidation on tuna polypeptide to determine optimal conditions. Ozonation efficiently deodorized tuna peptide powder. The optimal wet ozonation parameters for tuna peptides were as follows: ozone concentration of 99.1 mg/L, reaction time of 40 min, and temperature of 50 °C, yielding a sensory score of 2.2. This process maintained the protein composition and nutritional value of tuna peptide powder. Low-temperature stability tests revealed that the sensory score of ozonated tuna peptide powder in cosmetics remained stable, unaltered by temperature changes. These findings offer theoretical and practical insights for ozone deodorization in seafood processing, particularly in cosmetic applications. Unlike masking, adsorption, alkaline, or microbial-fermentation deodorization, ozone oxidation eliminates fishy aldehydes rapidly without introducing foreign volatiles, does not generate solid or liquid waste, and preserves both nitrogen content and sensory stability. These features make it particularly attractive for cosmetic formulations. However, applying ozone requires consideration of its chemical properties, treatment depth, safety, and equipment to ensure effective and safe deodorization. Comprehensive pre-implementation testing is essential to validate efficacy and preclude potential issues.

## Figures and Tables

**Table 1:** Sensory scores for samples in dry ozonation experiments (measured average value ± standard error, *n* = 3).

Experiment	Time (min)	Ozone Concentration (mg/L)	Sensory Score
Sensory scores for samples in single-factor dry ozonation experiments			
1	30	50.2	4.7 ± 0.2 ^a^
2	60	50.2	4.7 ± 0.2 ^a^
3	90	50.2	4.5 ± 0.1 ^ab^
4	120	50.2	4.1 ± 0.2 ^bc^
5	180	50.2	4.1 ± 0.1 ^bc^
6	240	50.2	4.0 ± 0.1 ^bcd^
7	300	50.2	4.0 ± 0.1 ^bcd^
8	60	0.1	5.0 ± 0.2 ^a^
9	60	4.2	4.8 ± 0.3 ^a^
10	60	50.2	4.5 ± 0.3 ^ab^
11	60	99.1	3.7 ± 0.3 ^cd^
12	60	104.6	3.5 ± 0.4 ^d^
Sensory scores for samples in the orthogonal dry ozonation experiments			
1	60	60.8	4.1 ± 0.3 ^ab^
2	60	99.1	4.2 ± 0.4 ^a^
3	60	101.2	3.8 ± 0.4 ^ab^
4	120	60.8	3.6 ± 0.3 ^ab^
5	120	99.1	3.8 ± 0.4 ^ab^
6	120	101.2	3.7 ± 0.3 ^ab^
7	180	60.8	3.6 ± 0.4 ^ab^
8	180	99.1	3.4 ± 0.2 ^b^
9	180	101.2	3.5 ± 0.3 ^ab^

^a–d^: Significant differences among samples (*p* < 0.05).

**Table 2:** Sensory scores for samples in the wet ozonation experiments (measured average value ± standard error, *n* = 3).

Experiment	Time (min)	Temperature (°C)	Ozone Concentration (mg/L)	Sensory Score
Sensory scores for samples in the single-factor wet ozonation experiments				
1	30	35	50.2	4.6 ± 0.1 ^ab^
2	60	35	50.2	4.6 ± 0.1 ^ab^
3	90	35	50.2	4.5 ± 0.1 ^bc^
4	120	35	50.2	4.3 ± 0.2 ^c^
5	180	35	50.2	4.3 ± 0.1 ^c^
6	240	35	50.2	4.4 ± 0.1 ^bc^
7	300	35	50.2	4.5 ± 0.1 ^bc^
8	60	35	0.1	5.0 ± 0.3 ^a^
9	60	35	4.2	4.8 ± 0.3 ^ab^
10	60	35	50.2	4.5 ± 0.3 ^bc^
11	60	35	99.1	2.2 ± 0.3 ^e^
12	60	35	104.6	2.8 ± 0.2 ^d^
13	60	30	50.2	4.2 ± 0.2 ^c^
14	60	40	50.2	4.2 ± 0.1 ^c^
15	60	50	50.2	4.0 ± 0.2 ^c^
16	60	60	50.2	4.2 ± 0.2 ^c^
17	60	70	50.2	4.4 ± 0.3 ^bc^
Sensory scores for the samples in the orthogonal wet ozonation experiments				
1	20	30	50.2	4.6 ± 0.3 ^a^
2	30	35	50.2	4.4 ± 0.3 ^ab^
3	40	40	50.2	4.7 ± 0.3 ^a^
4	50	45	50.2	4.3 ± 0.3 ^abc^
5	60	50	50.2	4.4 ± 0.3 ^ab^
6	20	35	60.8	3.7 ± 0.3 ^bcd^
7	30	40	60.8	3.6 ± 0.1 ^cde^
8	40	45	60.8	3.6 ± 0.1 ^cde^
9	50	50	60.8	3.5 ± 0.4 ^def^
10	60	30	60.8	3.6 ± 0.5 ^cde^
11	20	40	99.1	3.4 ± 0.2 ^defg^
12	30	45	99.1	2.9 ± 0.2 ^efghi^
13	40	50	99.1	2.3 ± 0.3 ^i^
14	50	30	99.1	2.6 ± 0.3 ^hi^
15	60	35	99.1	2.8 ± 0.3 ^fghi^
16	20	45	101.2	3.1 ± 0.3 ^defgh^
17	30	50	101.2	2.8 ± 0.3 ^fghi^
18	40	30	101.2	2.9 ± 0.3 ^efghi^
19	50	35	101.2	2.7 ± 0.3 ^ghi^
20	60	40	101.2	3.1 ± 0.3 ^defgh^
21	20	50	104.6	3.2 ± 0.2 ^defgh^
22	30	30	104.6	2.8 ± 0.4 ^fghi^
23	40	35	104.6	2.8 ± 0.5 ^fghi^
24	50	40	104.6	2.7 ± 0.4 ^ghi^
25	60	45	104.6	2.7 ± 0.4 ^ghi^

^a–i^: Significant differences among samples (*p* < 0.05).

**Table 3:** Effects of dry and wet ozonation on total nitrogen and amino nitrogen content of tuna peptide powder (measured average value ± standard error, *n* = 3).

	Original Sample	Dry Sample	Wet Sample
Total nitrogen content (calculated as N) (g/100 g)	15.4 ± 0.3 ^a^	14.3 ± 0.1 ^b^	15.0 ± 0.2 ^a^
Amino acid nitrogen (g/100 g)	2.5 ± 0.1 ^a^	2.2 ± 0.2 ^a^	2.3 ± 0.1 ^a^

^a^ and ^b^: Significant differences among samples (*p* < 0.05).

**Table 4:** Sensory scores for cosmetic samples during low-/high-temperature stability tests (moderately acceptable sensory score is 2–3, barely acceptable sensory score is 3–4, and unacceptable sensory score is 4–5).

Sample	Sample Returned to Room Temperature Within				
	1 Day	3 Days	7 Days	21 Days	28 Days
Cosmetic containing 1% untreated tuna peptide powder	Barely acceptable	Unacceptable	Unacceptable	Unacceptable	Unacceptable
Cosmetic containing 1% ozone-treated tuna peptide powder	Moderately acceptable	Moderately acceptable	Moderately acceptable	Moderately acceptable	Moderately acceptable

**Table 5:** Changes in the quality of fishy volatiles before and after oxidation (measured average value ± standard error, *n* = 3, *p* < 0.05).

Volatile Substance	Original Concentration (µg/L)	Concentration of Treated Sample (µg/L)	OT (µg/L)	The OAV of Original Sample	The OAV of Treated Sample	Reduction Level (%)
n-Hexaldehyde	5.9 ± 0.2	2.0 ± 0.1	0.02	295	100	66.5 ± 0.2
Nonaldehyde	0.8 ± 0.1	1.9 ± 0.2	1.2	0.7	1.6	−59.3 ± 0.1
Octanal	0.3 ± 0.2	0.5 ± 0.1	5.0	0.06	0.1	−33.3 ± 0.1

OT: Odor Threshold, OAV: Odor Activity Value.

**Table 6:** Retention time and selected ion monitoring mass parameters of three compounds.

Volatile Substance	Retention Time (min)	Qualitative Factor (*m*/*z*)	Categorical Factor (*m*/*z*)
n-Hexaldehyde	19.797	57	71
Nonaldehyde	16.080	57	43
Octanal	12.238	57	83

## Data Availability

The original data presented in the study are included in the article; further inquiries can be directed to the corresponding author.

## Supplementary Materials

The following supporting information can be downloaded at: https://www.mdpi.com/article/10.3390/md23050191/s1, Figures S1–S16: GC-MS results of samples before and after treatment, and the results for different compounds before and after treatment; Table S1: Peak areas of selected compounds in tuna peptide powder before and after ozone treatment.
