# Supplementary material for: Improving the Value Utilization of Tuna Peptide Powder for the Cosmetics Field Through Ozone Oxidation
Source: Mar Drugs. 2025 Apr 28;23(5):191. doi: 10.3390/md23050191 (PMC12113188; doi:10.3390/md23050191)
Supplement: Supplementary file 1 [file marinedrugs-23-00191-s001.zip › marinedrugs-3574841-supplementary.pdf]

Table S1. Peak areas of selected compounds in tuna peptide powder before and after ozone treatment.

	Compounds	Characteristic ions(m/z)	Peak area	
			Original sample	Treated sample
**Alcohol**	**2-Ethyl-1-hexanol**	112	607,765	161,583
**Ester**	**Butyl butyrate**	71	881,103	314,209
**Carbonyl compounds**	**Benzaldehyde**	105	4,593,146	11,309,596
**Carbonyl compounds**	**3-Heptanone**	72	472,265	137,964
**Hydrocarbon**	**Tetradecane**	57	20,796,327	1,760,293
**Sulfur-containing compounds**	**Dimethyl trisulfide**	126	5,307,307	1,945,782
**Others**	**Dibutyl phthalate**	149	1,238,331	7,095,252

Figure S1. The main compounds in the original sample.

Figure S2. The main compounds in the treated sample.

Figure S3. The 2-ethyl-1-hexanol in the original sample.

Figure S4. The 2-ethyl-1-hexanol in the treated sample.

Figure S5. The butyl butyrate in the original sample.

Figure S6. The butyl butyrate in the treated sample.

Figure S7. The benzaldehyde in the original sample.

Figure S8. The benzaldehyde in the treated sample.

Figure S9. The 3-heptanone in the original sample.

Figure S10. The 3-heptanone in the treated sample.

Figure S11. The tetradecane in the original sample.

Figure S12. The tetradecane in the treated sample.

Figure S13. The dimethyl trisulfide in the original sample.

Figure S14. The dimethyl trisulfide in the treated sample.

Figure S15. The dibutyl phthalate in the original sample.

Figure S16. The dibutyl phthalate in the treated sample.

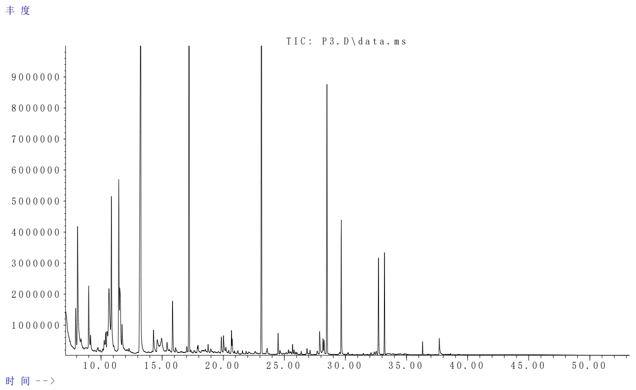

Figure S1. The main compounds in the original sample.

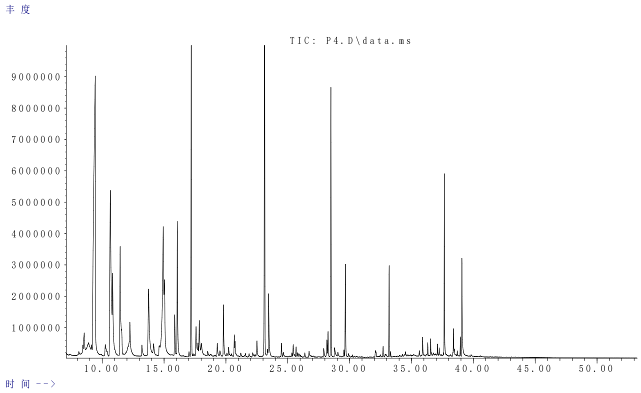

Figure S2. The main compounds in the treated sample.

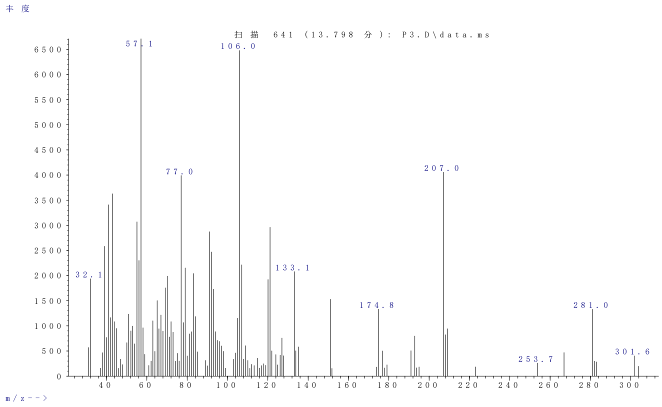

Figure S3. The 2-ethyl-1-hexanol in the original sample.

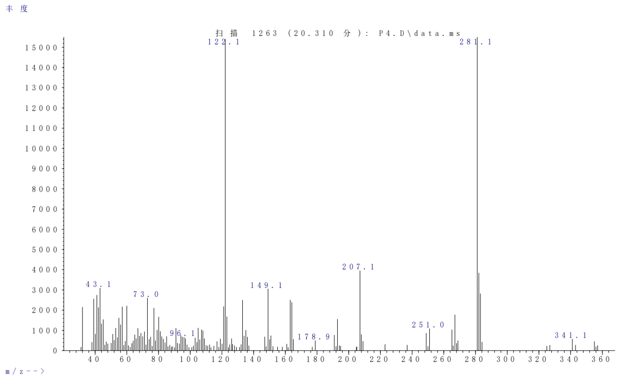

Figure S4. The 2-ethyl-1-hexanol in the treated sample.

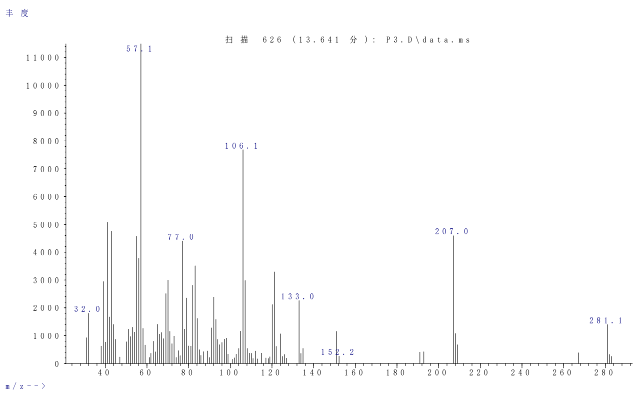

Figure S5. The butyl butyrate in the original sample.

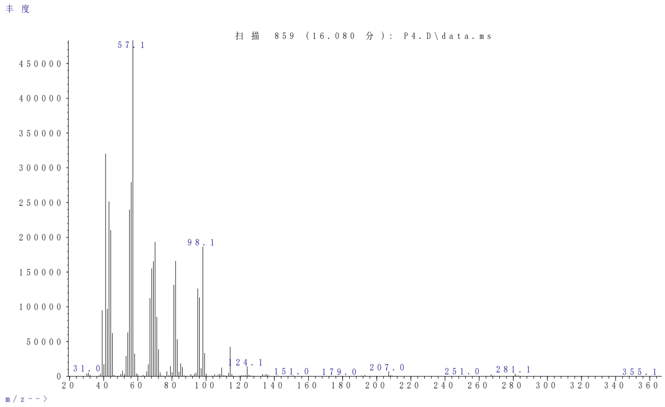

Figure S6. The butyl butyrate in the treated sample.

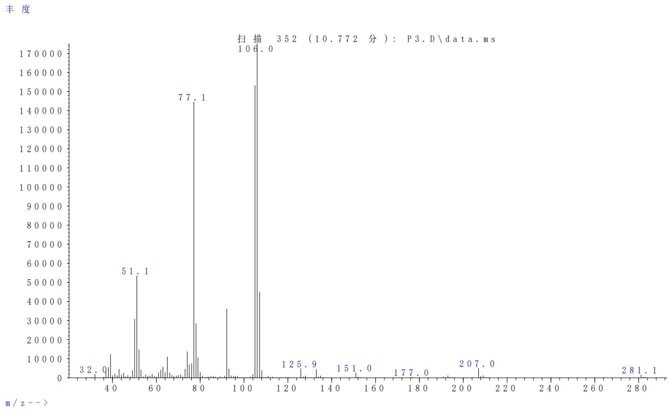

Figure S7. The benzaldehyde in the original sample.

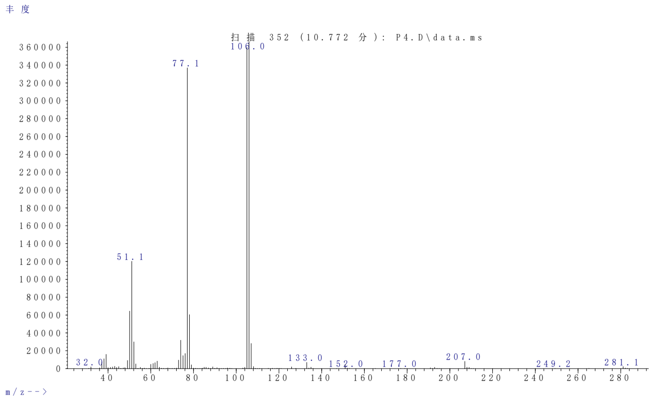

Figure S8. The benzaldehyde in the original sample.

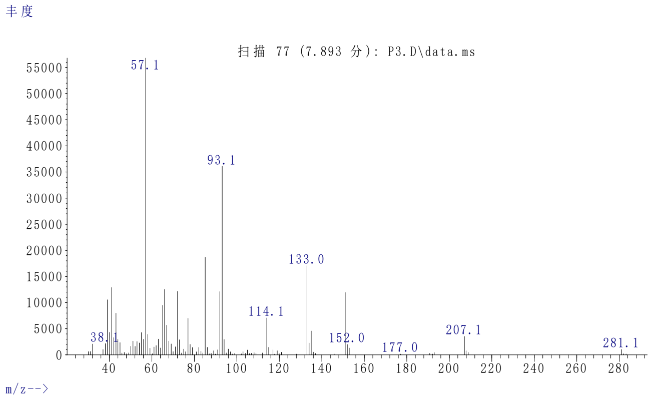

Figure S9. The 3-heptanone in the original sample.

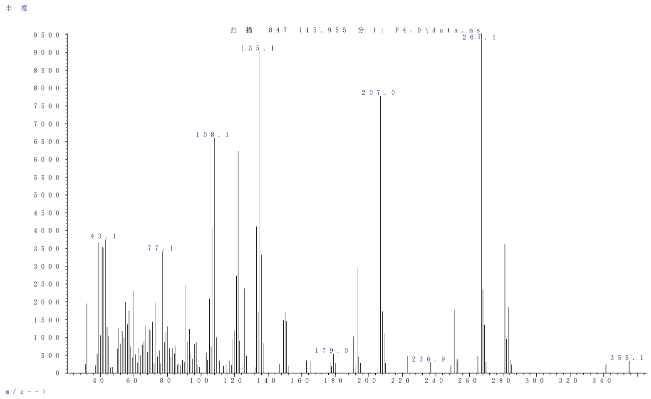

Figure S10. The 3-heptanone in the treated sample.

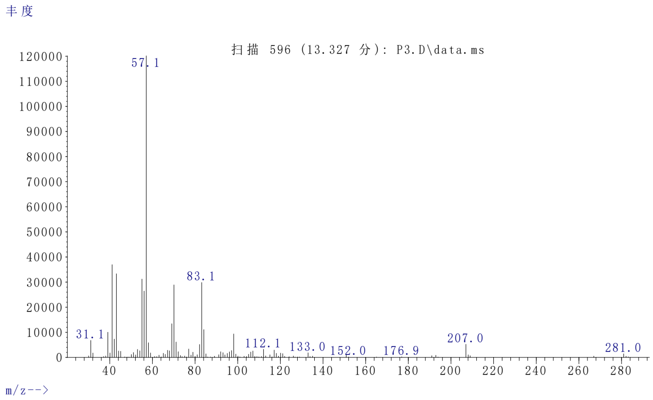

Figure S11. The tetradecane in the original sample.

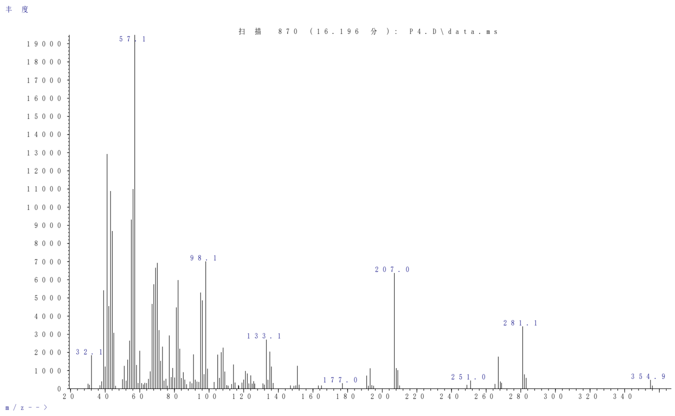

Figure S12. The tetradecane in the treated sample.

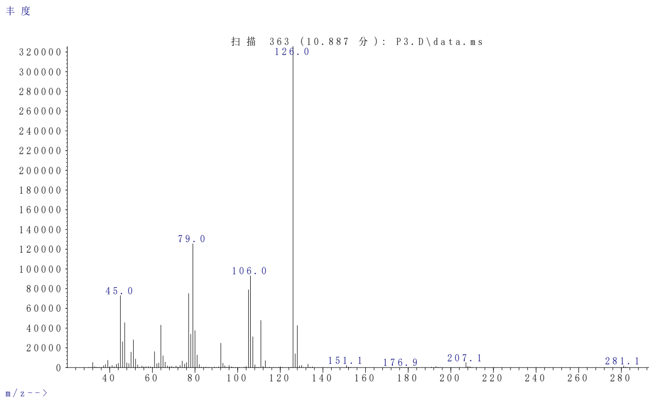

Figure S13. The dimethyl trisulfide in the original sample.

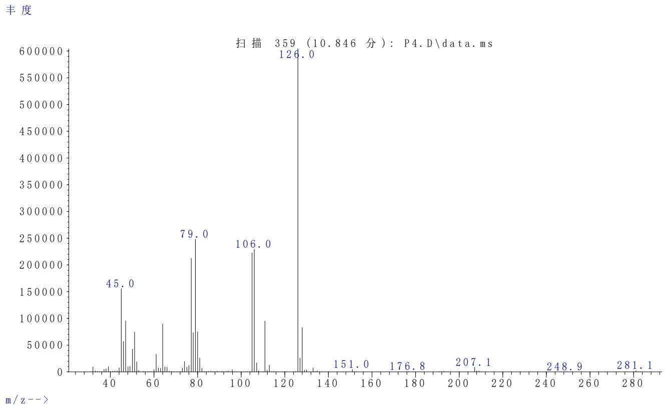

Figure S14. The dimethyl trisulfide in the treated sample.

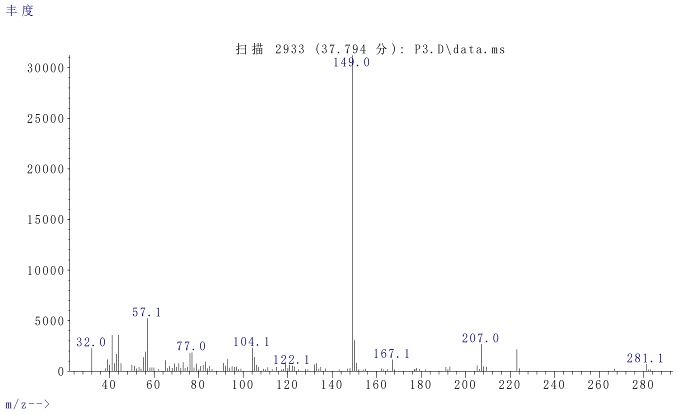

Figure S15. The dibutyl phthalate in the original sample.

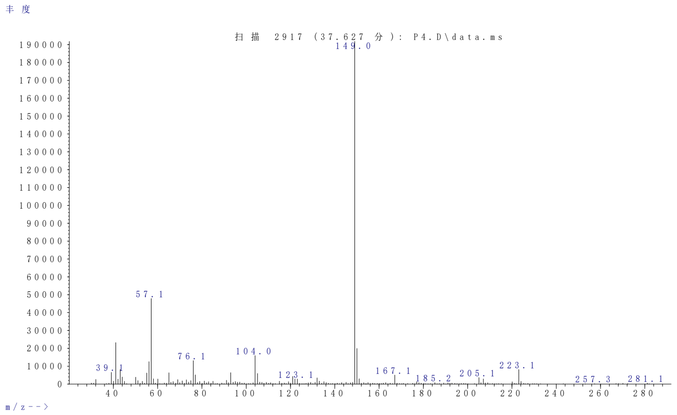

Figure S16. The dibutyl phthalate in the treated sample.
